# Paediatric burns secondary to nail adhesives: a case series

**DOI:** 10.1186/s41038-016-0048-6

**Published:** 2016-09-05

**Authors:** Claire Coles, Muhammad Umair Javed, Sarah Hemington Gorse, Dai Nguyen

**Affiliations:** Welsh Centre for Burns and Plastic Surgery, Morriston Hospital, Swansea, SA6 6NL UK

**Keywords:** Nail adhesive, Nail glue, Chemical burn, First aid, Paediatric burns

## Abstract

**Background:**

Nail adhesives are widely available beauty products that are used in the cosmetic industry and households to attach false nails. Nail adhesive burns are uncommon, and it is crucial that healthcare providers and the public are aware of its potential to cause chemical burn.

**Case Presentation:**

Case series of accidental burns secondary to cyanoacrylate nail glue treated at the Welsh Centre for Burns and Plastic Surgery (WCBPS) in Swansea, United Kingdom.

**Conclusion:**

All of the burns were observed in children and occurred due to accidental spillage. Therefore, it is important for the public to be aware that nail glue is a chemical agent which should be stored safely away from the reaches of young children. The case series highlights the importance of first aid in reducing the severity of chemical burns secondary to the nail adhesives, and its early recognition and treatment are emphasised.

## Background

Nail adhesives are widely available beauty products used in both the cosmetic industry and households to attach false nails. The primary ingredient of the nail glue is cyanoacrylate. Cyanoacrylate is an acrylic resin that, in the presence of moisture, rapidly sets. There are several reports published in the literature and media documenting burn injuries following its accidental spillage [1–7]. We report a case series of burn injuries secondary to nail glue use treated at the Welsh Centre for Burns and Plastic Surgery. The purpose of this case series is to raise the awareness of its potential to cause burn injuries and to reiterate the importance of first aid in reducing the severity of the burn and its early recognition and treatment.

## Case presentation

### Case 1

**Fig. 1:**
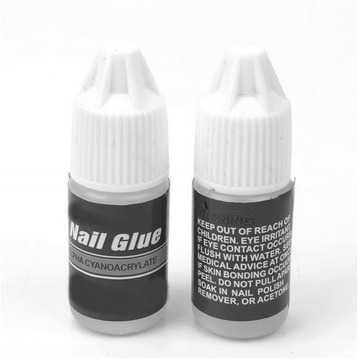
Nail adhesive used in case 1. Please note the absence of risk of burn injury on the warning label

**Fig. 2:**
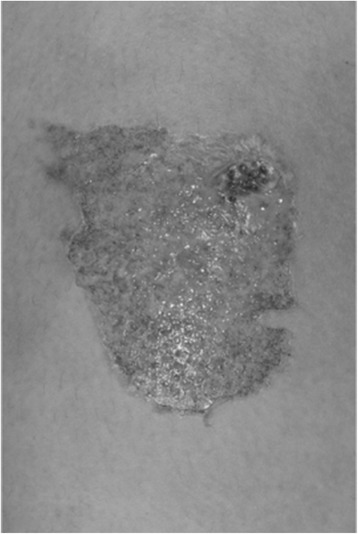
Total body surface area (TBSA) (0.5 %) mixed depth burn on the anterior aspect of thigh. The patient was initially managed conservatively before undergoing surgery

A 15-year-old girl was referred to our centre at day 3 post-burn to her thigh. She dropped a small amount of nail glue onto her thigh when she had been applying nail glue on to her fake nails (Fig. 1). The nail glue remained in contact for a total of 5 min before she felt the burning sensation. She then removed her trousers and noticed the burns. No first aid was instituted. She attended the local emergency department (ED) on the same day. At her initial assessment, the wound was irrigated for 30 min with cold water and dressed, then she was discharged home. However, she returned to the ED 2 days later due to increasing erythema around the burn. She was started on oral antibiotics and was referred to the burns centre. On examination, a 0.5 % total body surface area (TBSA) burn of mixed depth was observed on the upper medial aspect of her right thigh (Fig. 2), and it was approximately 7 × 5 cm in size. She was initially managed with Mepilex® Ag (Molnlycke Health Care, Gothenburg, Sweden) and then with flamazine cream and daily dressings.

She was reviewed a week later, and due to the delayed wound healing, she was counselled to proceed with surgical excision of the burn and reconstruction with split-thickness skin grafting. The patient underwent surgery and was discharged home the same day. The graft was successful and healed completely without complications within 2 weeks.

## Case 2

A three-and-a-half-year-old fit and well girl presented to our centre with a burn on the right hand. She had taken a bottle of nail glue out of her aunt’s handbag and tried to open the bottle with her teeth. The glue spilled onto the child’s right hand sleeve causing the sleeve to adhere to her arm. The patient’s mother immediately removed the child’s clothing revealing an area of blistered skin. The burn was irrigated under cold water. She presented to the local hospital and was referred immediately to our burns centre.

On examination, she had approximately 0.5 % TBSA burn across the dorsum of the right forearm and the right hand with some blisters. An area of dried glue to the back of the hand was also noted. The burns were managed conservatively with Mepilex® Lite (Molnlycke Health Care, Gothenburg, Sweden) and healed without complication.

### Case 3

**Fig. 3:**
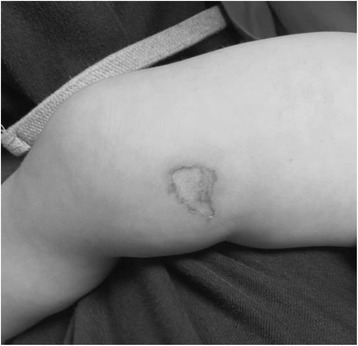
A one-and-half-year-old patient with 0.25 % TBSA full-thickness burn on the medial aspect of the right thigh. The burn was managed conservatively

A one-and-a-half-year-old girl presented to a local hospital after accidently spilling a bottle of nail glue on her thigh. Her mother had immediately removed the child’s pyjamas and irrigated her leg under tap water before attending the accident and emergency department. On examination, she had an approximately 0.25 % TBSA full-thickness burn on the right inner thigh (Fig. 3). The skin was pale and insensate. Due to the small percentage of the burn, it was treated conservatively with dressings. At the fifth week follow-up, the burn had healed satisfactorily and the patient was discharged with a planned review by our burns outreach team.

### Case 4

A 2-year-old boy presented to a local hospital with spillage of nail glue over his hand, abdomen, thigh and knee. His mother had immediately removed the patient’s clothes and irrigated under tap water for few minutes. On examination, the glue had peeled off completely over the contact areas on the left hand, knee, thigh and left side of abdomen, but no burn injury was identified. Hence, his mother was reassured and the patient was discharged.

### Discussion

Chemical burn is an uncommon cause of burn injuries in the paediatric population and accounts for less than 10 % of burns treated in the emergency department [8].

Cyanoacrylates are powerful adhesives and represent the primary ingredient of nail glue. Nail glue can cause localised dermatitis, paronychia and allergic onycholysis on skin contact. Chemical burns secondary to nail glue are uncommon [9, 10].

When in contact with moisture, the cyanoacyclates react and cause an exothermic reaction which can lead to a burn [6]. Tang et al., in their case report, suggested a similar mechanism for the burn injury, with moisture in a patient’s denim jean as the trigger for exothermic reaction. However, other authors have reported burns after direct contact of the adhesive with the skin [7]. In our case series, all but one of the cases reported contact of the adhesive with the clothing eventually causing the burn injury.

Often, the chemical burn due to adhesive is not instant; therefore, the patient is unaware and does not take an immediate action. A key component of managing chemical burns is to remove all traces of the chemical including the affected clothing and thoroughly irrigating the area. If the clothing is strongly adherent to the skin after spillage, pulling the clothing off should be avoided and soaking the affected area with tap water is often helpful. Monitoring the pH of the wound, although it is impractical outside a hospital setting, may help to determine the adequacy of the irrigation [8].

In the literature, concern has been raised regarding the inadequate labelling of nail glue products, which fail to highlight the risk of burn injury [9]. Though several commercially available nail glues do have a warning of risk of burn injury on their labels, the product used in case 1 did not warn against a burn injury (Fig. 1).

All of the burns were observed in a paediatric population and occurred due to accidental spillage. Therefore, it is important for the public to be aware that nail glue is a chemical agent which should be stored safely away from the reaches of young children. In addition, non-accidental injury should be excluded in all paediatric burn injuries. In the cases presented, the clinical findings were consistent with the circumstances and the history of the injury.

## Conclusions

Nail adhesive burns are uncommon and hence, it is crucial that healthcare providers and the public should be aware of the potential of nail adhesives to cause chemical burn. All of the burns were observed in children and occurred due to accidental spillage. Therefore, it is important for the public to be aware that nail glue is a chemical agent which should be stored safely away from the reaches of young children. The case series highlights the importance of first aid in reducing the severity of chemical burns secondary to the nail adhesives, and the early recognition and treatment are emphasised.

### Consent

Written informed consent was obtained from the patients for publication of this case report and any accompanying images. A copy of the written consent is available for review by the Editor-in-Chief of this journal.
